# Direct radiation-induced effects on dental hard tissue

**DOI:** 10.1186/s13014-019-1208-1

**Published:** 2019-01-11

**Authors:** Hui Lu, Qi Zhao, Jiang Guo, Binghui Zeng, Xinlin Yu, Dongsheng Yu, Wei Zhao

**Affiliations:** 1 Guanghua School of Stomatology, Hospital of Stomatology, Guangdong Provincial Key Laboratory of Stomatology, Sun Yat-sen University, Guangzhou, 510055 China; 2 Department of Oncology, Central Hospital of Xianning City, Tongji Xianning Hospital of Huazhong University of Science and Technology, Hubei, China; 3 International Department, The Affiliated High School of SCNU, Guangzhou, China

**Keywords:** Radiation, Dental hard tissue, Mechanical property, Crystal property, Micro-morphology, Chemical composition, DEJ

## Abstract

**Background:**

Radiation caries is a complication of radiotherapy characterized by enamel erosion and dentin exposure. The mechanisms of characteristic radiation caries formation are not well-understood. The aim of this study was to evaluate the direct radiation-induced effects on dental hard tissue and investigate their role in the formation of radiation caries.

**Methods:**

Sixty non-carious third molars were divided into three groups (*n* = 20), which would be exposed to 0 Gy, 30 Gy, and 60 Gy radiation, respectively. After radiation, microhardness and elastic modulus were measured at four depths by means of a Vickers microhardness tester and atomic force microscopy (AFM). The microstructure was observed by scanning electron microscopy (SEM). X-ray diffraction and Raman microspectroscopy were used to determine crystal properties and protein/mineral (2931/960 cm^− 1^) ratios.

**Results:**

A statistically significant decrease in microhardness and elastic modulus values 50 μm from the dentino-enamel junction (DEJ) in enamel was revealed in the 30-Gy and 60-Gy groups. With the increasing dose, destruction of interprismatic substance and fissures at the DEJ-adjacent region were found. A greater reduction of crystallinity was revealed in enamel compared with dentin. Raman spectroscopic analysis showed a slight increase of the protein/mineral ratio for enamel following accumulated radiation, while the protein/mineral ratio for dentin was decreased.

**Conclusions:**

Radiation could directly alter the mechanical properties, micro-morphology, crystal properties, and chemical composition of dental hard tissue. The early destruction of DEJ-adjacent enamel, combined with decreased crystallinity of enamel under radiation exposure, may be related to the formation of characteristic radiation caries.

## Background

Radiotherapy is widely used in the treatment of head and neck cancer. As one of the most threatening complications of radiotherapy, radiation caries exists at a high level of prevalence. A systematic review reported the mean prevalence of radiation caries to be 28.1%, and the mean average number of decayed, missing, and filled teeth (DMFT) of patients post-irradiation was 9.19 [1]. This kind of caries develops rapidly within a few months after radiation. Patients with radiation caries can develop periapical periodontitis or radiation osteomyelitis in some severe cases, with a high risk of dentition destruction [2, 3].

Typical radiation caries is characterized by enamel erosion and dentin exposure. It occurs mainly on labial surfaces at the cervical areas of teeth post-irradiation [4]. In addition to cervical areas, areas resistant to typical dental decay, such as occlusal and incisal edges of teeth, can be affected [5, 6]. The lesion is often noticed with shear fracture of enamel, followed by loss of enamel, exposing the underlying dentin. It is important that radiation caries differs in clinical appearance and patterns of onset and progression from caries in non-irradiated patients [7]. Additionally, since the structure constitutes a unique bonding between enamel and dentin, the dentino-enamel junction (DEJ) may play a crucial role in the pathological process of radiation caries.

Indirect effects of radiotherapy—including changes in salivary quantity and composition, together with alteration of the oral flora—are widely regarded as the major causes of radiation caries [8, 9]. However, these factors could not well explain the characteristic features of radiation caries, such as the initial loss of cervical and incisal enamel. In recent years, researchers have focused on the effects of direct radiation-induced damage on dental hard tissue [10, 11]. Though degenerative changes in the microhardness and microstructure of teeth were found [12], comprehensive assessments of direct radiation-induced impact on mineralized tooth substrates are still limited.

For a better understanding of the direct radiation-induced effects on dental hard tissue (including enamel, dentin, and the DEJ) and their role in the formation of post- irradiation dental lesions, the present study focused on characterizing the mechanical properties and micro-morphology, especially crystal properties and chemical composition of those tissues, in an attempt to elucidate the pathogenic mechanism of radiation caries.

## Methods

### Sample preparation and grouping

Sixty non-carious third molars extracted from 60 non-irradiated individuals were collected with informed consent at the Department of Oral and Maxillofacial Surgery in the Guanghua Hospital of Stomatology, Sun Yat-sen University. The exclusion criteria were the presence of fissures, enamel hypoplasia, and white spots. The study was approved by the Ethics Committee of Guanghua School of Stomatology, Sun Yat-sen University.

Teeth were randomly divided into three groups (*n* = 20): a 30-Gy group, a 60-Gy group, and a control group, which would subsequently be exposed to corresponding radiation doses. Teeth in each group were longitudinally sectioned into two slabs with a thickness of 2 mm, in a bucco-lingual direction. For each tooth, one slab would be designated for post-irradiation mechanical properties measurement, while the other slab was kept for post-irradiation histomorphological observation. All slabs were polished with 600-, 1200-, and 2000-grit SiC disks and rinsed ultrasonically with deionized water for 5 min.

### Irradiation procedure

Irradiation was carried out in the Sun Yat-sen University Cancer Center. Prior to irradiation, slabs were fixed with wax and located with the buccal surface upward. Slabs in the 30-Gy and the 60-Gy groups were irradiated in a linear accelerator (Elekta ELE1935, Stockholm, Sweden) with 6 MV photons. The source-surface distance was set at 100 cm. To simulate head and neck cancer radiotherapy, slabs in the two treated groups were exposed to fractional radiation (2 Gy/fraction/day, 5 days/week) to achieve a total dose of 30 Gy and 60 Gy, respectively. The control group was kept in saline without radiation exposure. After radiation, the dental slabs were rinsed with deionized water.

### Microhardness measurement

**Fig. 1:**
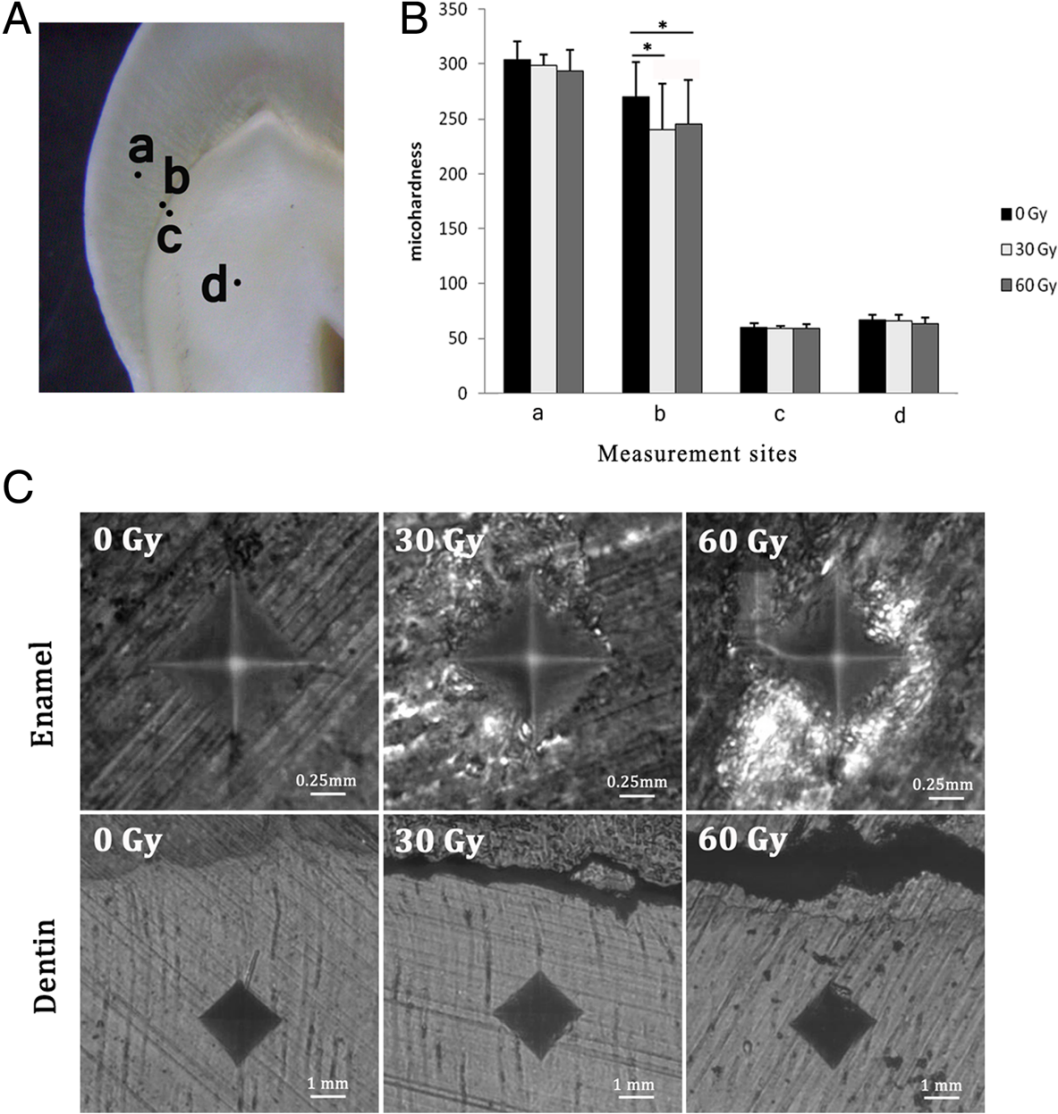
Vickers microhardness values and indentations of enamel and dentin in the three groups. **a** Example of an enamel slab showing the distribution of four measurement points. a, middle enamel; b, 50 μm from the DEJ in enamel; c, 50 μm from the DEJ in dentin; d, middle dentin. **b** Significant difference between the treated groups and the control group was found at the site 50 μm from the DEJ in enamel (point b). *p < 0.05. *n* = 20 in each; ANOVA. **c** Vickers indentations of the enamel and dentin

Microhardness measurement was performed on a Vickers microhardness tester (Struers DuraScan-20, Ballerup, Denmark), with a 25-gf load applied for 10 s in enamel and a 10-gf load for 15 s in dentin. Four sites on the dental longitudinal section surface were chosen and measured for each dental slab in the three groups (Fig. 1a). The four sites of dental slabs were set at one-half the thickness of buccal enamel (middle enamel), 50 μm from the DEJ in enamel, 50 μm from the DEJ in dentin, and one-half the thickness of dentin (middle dentin). For each site, three indentations were performed to obtain a mean microhardness value.

### Nanoindentation measurements and topographic analysis

Nanoindentation measurements for elastic modulus were carried out with a Berkovich tip on an atomic force microscope (AFM) (Bruker Dimension FastScan, Karlsruhe, Germany). Prior to measurement, specimens were irrigated ultrasonically for 10 min in deionized water and dried in a dryer at room temperature. Elastic modulus measurement was performed at the same four sites as those of microhardness measurements. For each site, three indentations were made. Force-displacement curves for each indentation were obtained, and the elastic modulus values of enamel, dentin, and the DEJ-adjacent region were calculated according to Oliver and Pharr’s equation in a contact mode [13] with NanoScope Analysis software (Bruker Optics, Inc.).

For topographic analysis, two slabs were selected from each group and etched with 0.1 mol/L citric acid for 1 min. The topography of the dental surface was observed with AFM over an area of 10 × 10 μm.

### Scanning electron microscopy and electron probe microanalysis

For scanning electron microscopy (SEM) and electron probe microanalysis (EPMA), four specimens from each group were fixed with 2.5% glutaraldehyde solution. After dehydration in increasing concentrations of ethanol solution (25, 50, 75, 95, and 100%), specimens were coated with a 20-nm gold-palladium layer. We used SEM (Philips XL30 FEG microscope, Eindhoven, The Netherlands) to observe the micro-morphological characteristics of enamel, dentin, and the DEJ in the three groups. Fissures in enamel were also analyzed by EPMA (JXA-8530F, JEOL, Tokyo, Japan) with area-mapping for the composition of the chemical elements Ca and P. X-ray profiles and element quantification were performed at 20 kV and 0.5 mA. The Ca/P ratios of the fissure and intact enamel were measured.

### Crystallographic assessments

Enamel and dentin from each group were ground into powder. XRD profiles of the samples were obtained from an x-ray diffractometer (PANalytical Empyrean, Almelo, The Netherlands) with the scanning angle (2*θ*) ranging from 5° to 65° at room temperature. The Cu-Kα radiation source (*λ* ≈ 0.15406 nm) was operated at 40 kV/40 mA. The phases and crystallinity of the enamel and dentin in each group were analyzed with Jade 5 software (MDI, Materials Data Inc., Livermore, CA, USA). The phases of the samples were identified based on the spectra of known phases from the Joint Committee on Powder Diffraction Standards (JCPDS). According to Scherrer’s formula, crystallinity can be calculated through the wavelength of the radiation source, the full-width half-maximum (FWHM), and the diffraction angle (*θ*); larger FWHM suggests lower crystallinity. Thus, the FWHM of each spectrum was calculated to reflect the crystallinity of enamel and dentin after different radiation doses [14].

### FT-Raman spectroscopy

Raman spectra of enamel powder and dentin powder in each group were acquired by Fourier Transform infrared Raman spectroscopy (HORIBA Jobin Yvon, Inc., Edison, NJ, USA) with a near-infrared (785 nm) laser. The spectrum data were collected over the range of 3700–400 cm^− 1^ with spectral resolution of 4 cm^− 1^. Spectral deconvolution was performed with Labspec 5 software (HORIBA Jobin Yvon, Inc.). After a polynomial baseline correction to remove the background due to fluorescence, areas under the bands at 960 cm^− 1^ and 2931 cm^− 1^ were determined, for analysis of differences in mineral and protein compositions in both enamel and dentin. Based on the Raman spectral data, the ratios of protein at 2931 cm^− 1^ to phosphate at 960 cm^− 1^ were calculated.

### Statistical analysis

Continuous variables (microhardness and elastic modulus values) with normal distribution and equal variances were analyzed with analysis of variance (ANOVA) and expressed as means ± SD. Statistical differences in the microhardness and elastic modulus were analyzed with SPSS 22 software (IBM, Armonk, NY, USA). The significance level was set at 5%.

## Results

### Microhardness analysis

At the site 50 μm from the DEJ in enamel, identified as point ‘a’ in Fig. 1a, the microhardness values in the 30-Gy and the 60-Gy groups were obviously lower than those in the control group (*p* < 0.05). However, at the sites of middle enamel, middle dentin, and 50 μm from the DEJ in dentin, there was no significant difference in the microhardness values among the three groups (Table 1, Fig. 1b). The findings of indentation images were consistent with the results of statistical analysis. As can be seen in Fig. 1c, the indentation of the Vickers indenter was square. With the dose increasing, more fragments were found around the indentation. Fissures in enamel near the DEJ were also present.

**Table 1:** Vickers microhardness values of enamel and dentin in the three groups

	Middle enamel (point a)	DEJ + 50 μm (point b)	DEJ – 50 μm (point c)	Middle dentin (point d)
0 Gy	303.47 ± 17.35	270.22 ± 31.27 ^▲●^	59.75 ± 4.20	66.57 ± 5.20
30 Gy	298.55 ± 9.96	240.12 ± 42.20 ^▲^	59.75 ± 4.20	66.28 ± 5.44
60 Gy	293.08 ± 20.09	245.37 ± 39.81^●^	59.62 ± 3.55	63.78 ± 5.59

*DEJ + 50 μm* = 50 μm from the DEJ in enamel. *DEJ – 50 μm =* 50 μm from the DEJ in dentin

The position of point a, b, c and d is showed in Fig. 1a

Identical symbols (^▲●^) denote statistically significant difference

### Nanoindentation measurements and topographic analysis

**Fig. 2:**
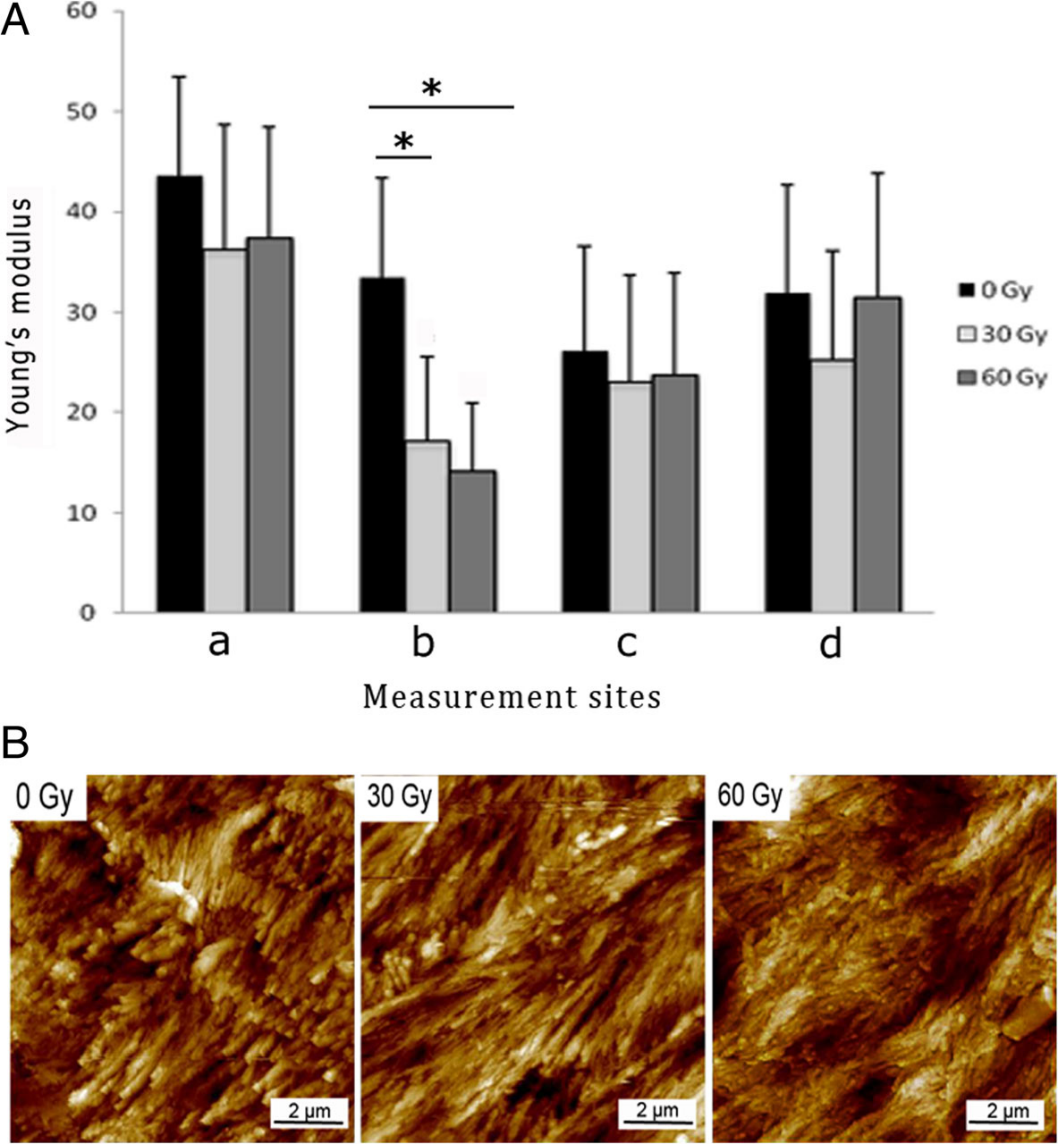
Elastic modulus values and topography of enamel and dentin in the three groups, determined by AFM. **a** Elastic modulus measurement was performed at the same four sites as those of microhardness measurements. a, middle enamel; b, 50 μm from the DEJ in enamel; c, 50 μm from the DEJ in dentin; d, middle dentin. At the site 50 μm from the DEJ in enamel (point b), Young’s modulus values in the 30-Gy and 60-Gy groups were significantly lower than in the control. *p < 0.05. n = 20 in each; ANOVA. **b** Typical topographic mapping of enamel. With the dose increasing, more diffused enamel prism and impaired interprismatic substance can be seen

The trend of elastic modulus values in the three groups was similar to that of microhardness (Table 2, Fig. 2a). For the site 50 μm from the DEJ in enamel, the elastic moduli in the 30-Gy and 60-Gy groups were significantly lower than that of the control (*p* < 0.05). However, no statistically significant difference was found among the three groups at the sites of middle enamel, middle dentin, and 50 μm from the DEJ in dentin (*p* > 0.05).

**Table 2:** Elastic modulus values (GPa) of enamel and dentin in the three groups

	Middle enamel (point a)	DEJ + 50 μm (point b)	DEJ – 50 μm (point c)	Middle dentin (point d)
0 Gy	43.57 ± 9.81	33.36 ± 9.96 ^▲●^	26.12 ± 10.46	31.88 ± 10.82
30 Gy	36.16 ± 12.57	17.02 ± 8.58 ^▲^	23.00 ± 10.70	25.22 ± 10.84
60 Gy	37.36 ± 11.05	14.01 ± 6.96^●^	23.64 ± 10.29	31.45 ± 12.36

*DEJ + 50 μm* = 50 μm from the DEJ in enamel. *DEJ – 50 μm =* 50 μm from the DEJ in dentin

The position of point a, b, c and d is showed in Fig. 1a

Identical symbols (^▲●^) denote statistically significant difference

Topographic analysis by AFM revealed an impaired interprismatic substance in the treated groups (Fig. 2b). After radiation, enamel rods were shortened and irregularly arranged. In the 60-Gy group, erosion of enamel prism was obvious, and the prismatic structure had become amorphous and hard to recognize.

### SEM and EPMA observations

**Fig. 3:**
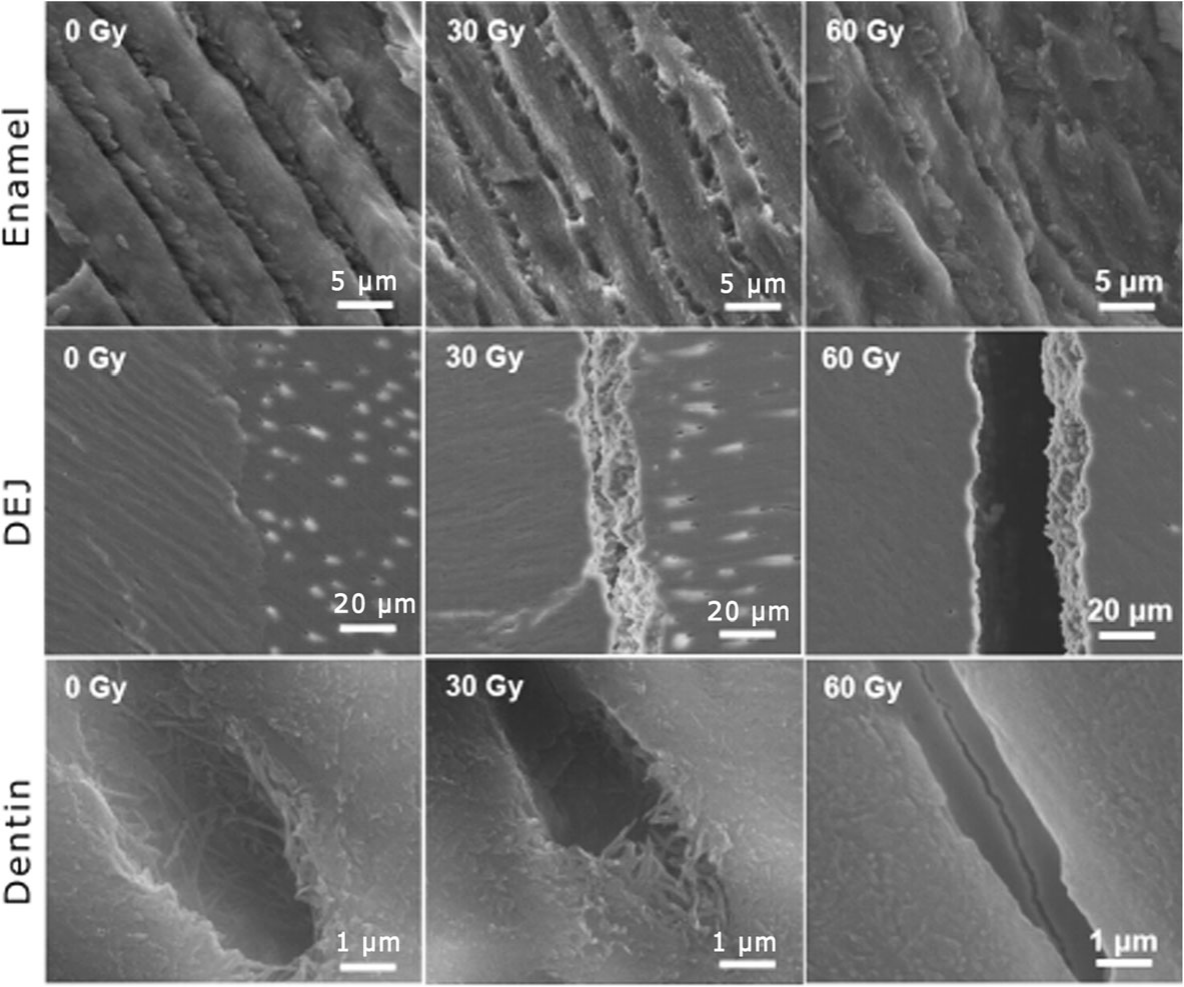
SEM micrographs of enamel, dentin, and the DEJ in the three groups. With the dose increasing, progressive destruction of interprismatic substance and enamel prism was revealed. Fragments of enamel prism were evident in the 30-Gy and 60-Gy groups, and the arrangement of the enamel prism was irregular. Fissures at the DEJ were obvious in the treated groups. In dentin, obliterated dentinal tubules, degeneration of the collagen network, and cracks on the tubular walls can be seen in the 30-Gy and 60-Gy groups

Upon scanning electron microscopy (SEM) observation, well-defined enamel prism, dentinal tubules, and the DEJ were displayed in the control group. A progressive destruction of interprismatic substance and enamel prism was revealed with increasing doses. Fragments of enamel prism were evident in the 30-Gy and 60-Gy groups, and the arrangement of the enamel prism was irregular. In dentin, obliterated dentinal tubules, degeneration of the collagen network, and cracks on the tubular wall could be seen in the 30-Gy and 60-Gy groups. Fissures at the DEJ in the treated groups were obvious with the increasing doses (Fig. 3).

**Fig. 4:**
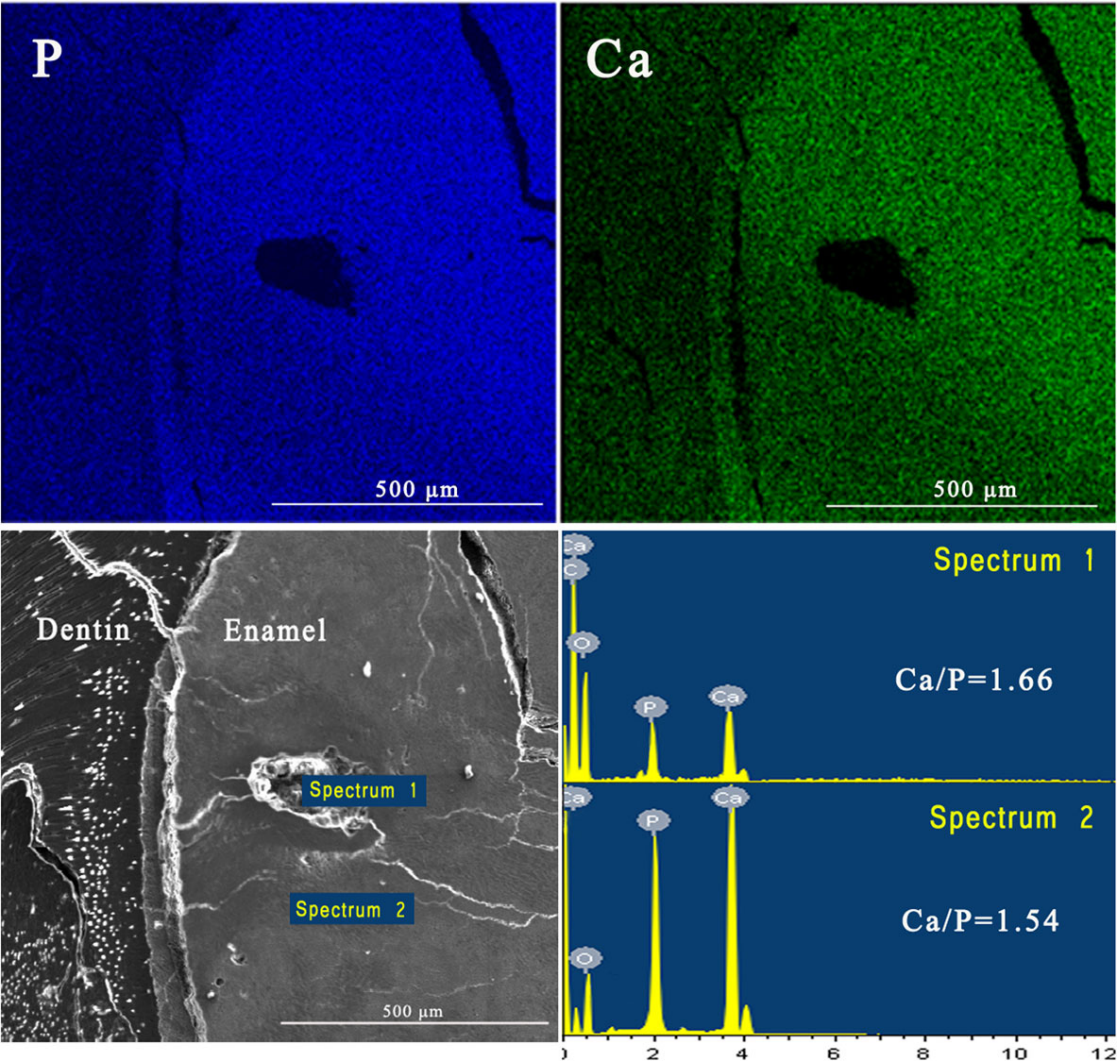
Representative EPMA mapping for Ca and P over the fissure. The Ca and P contents of the enamel without fissure were visibly higher than that of the fissure. The Ca/P ratio in the fissure was higher than that of the intact enamel, indicating loss of calcium-deficient apatite in the fissure

Representative electron probe microanalysis (EPMA) mapping for Ca and P over the fissure near the DEJ is shown in Fig. 4; energy-dispersive spectra for relatively intact enamel and the fissure are also exhibited. The Ca and P contents of the enamel without a fissure appear clearly higher than that of the fissure. The Ca/P ratio of the enamel without a fissure was 1.54, while the Ca/P ratio in the fissure was 1.66, close to the Ca/P ratio of hydroxyapatite [Ca_10_(PO_4_)_6_(OH)_2_].

### Crystallinity evaluation of enamel and dentin apatite

**Fig. 5:**
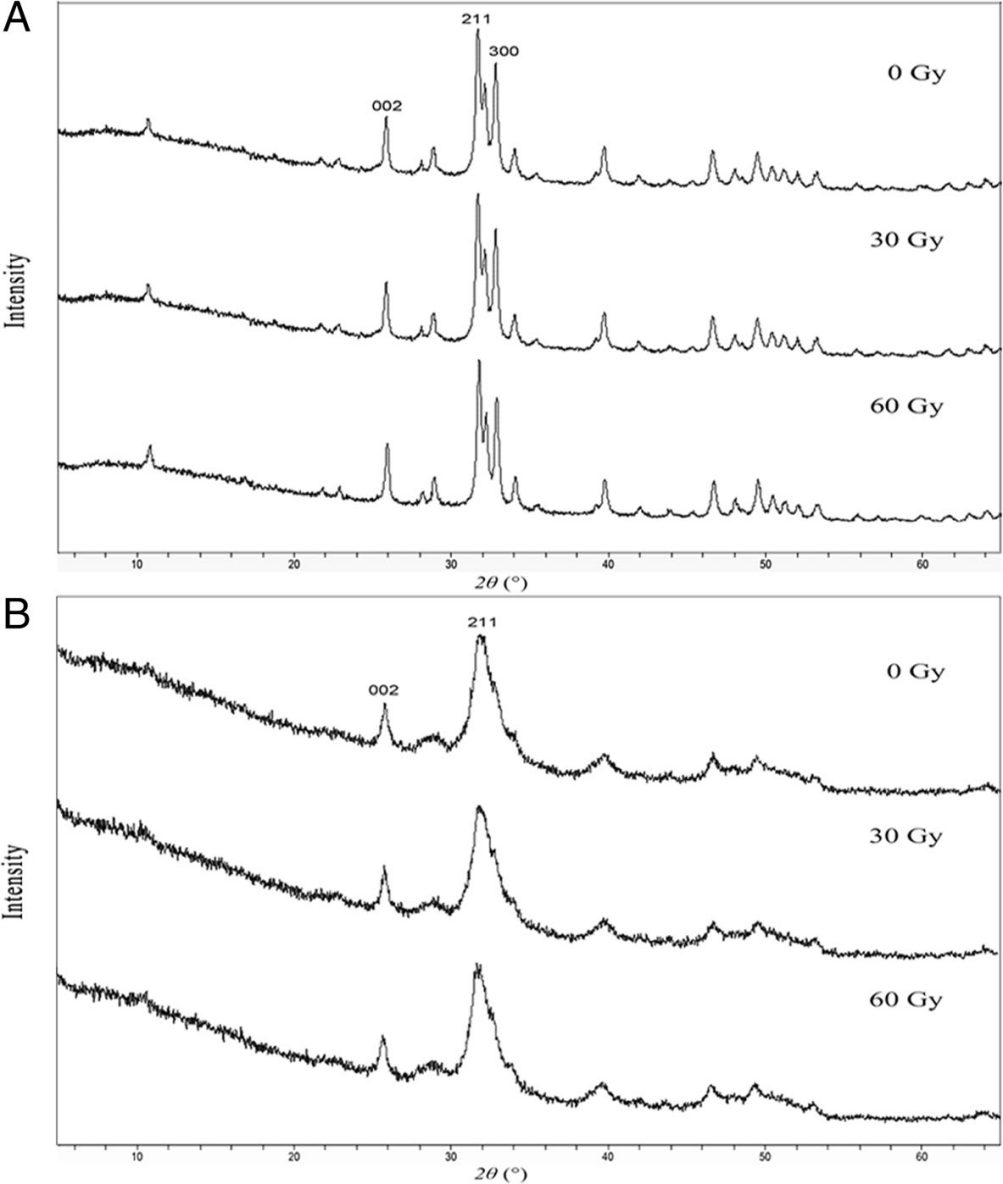
XRD patterns of **a** enamel and **b** dentin in the three groups. The enamel and the dentin were composed of various amounts of hydroxyapatite [HA, Ca_10_(PO_4_)_6_(OH)_2_] and calcium-deficient apatite [Ca_10-x_(HPO_4_)_x_(PO_4_)_6-x_(OH)_2-x_, x = 0.5–1.3]. Based on the Joint Committee on Powder Diffraction Standards (JCPDS) cards, no new phase was detected in the 30-Gy and 60-Gy groups

XRD analysis (Fig. 5) revealed the major phase of enamel in the three groups to be hydroxyapatite [HA, Ca_10_(PO_4_)_6_(OH)_2_]. Moreover, there was a small amount of calcium-deficient apatite [Ca_10-x_(HPO_4_)_x_(PO_4_)_6-x_(OH)_2-x_, x = 0.5–1.3]. Based on Joint Committee on Powder Diffraction Standards (JCPDS) cards, no new phase was detected in the 30-Gy and 60-Gy groups. The FWHM of enamel was gradually enlarged with increased radiation doses, ranging from 0.295 to 0.315. The FWHM of dentin in the three groups was also enlarged, ranging from 0.847 to 0.859, with an appearance of less-sharp peaks in XRD patterns (Table 3). Compared with dentin, enamel revealed more enlargement of the FWHM, implying a greater reduction in crystallinity after the same radiation exposure.

**Table 3:** Comparison of crystallinity in the three groups

	Enamel		Dentin	
	FWHM	Enlargement	FWHM	Enlargement
0 Gy	0.295	NA	0.847	NA
30 Gy	0.309	4.75%	0.854	0.83%
60 Gy	0.315	6.78%	0.859	1.42%

*FWHM* full-width half-maximum; *NA* not applicable

### Raman microspectroscopy

**Fig. 6:**
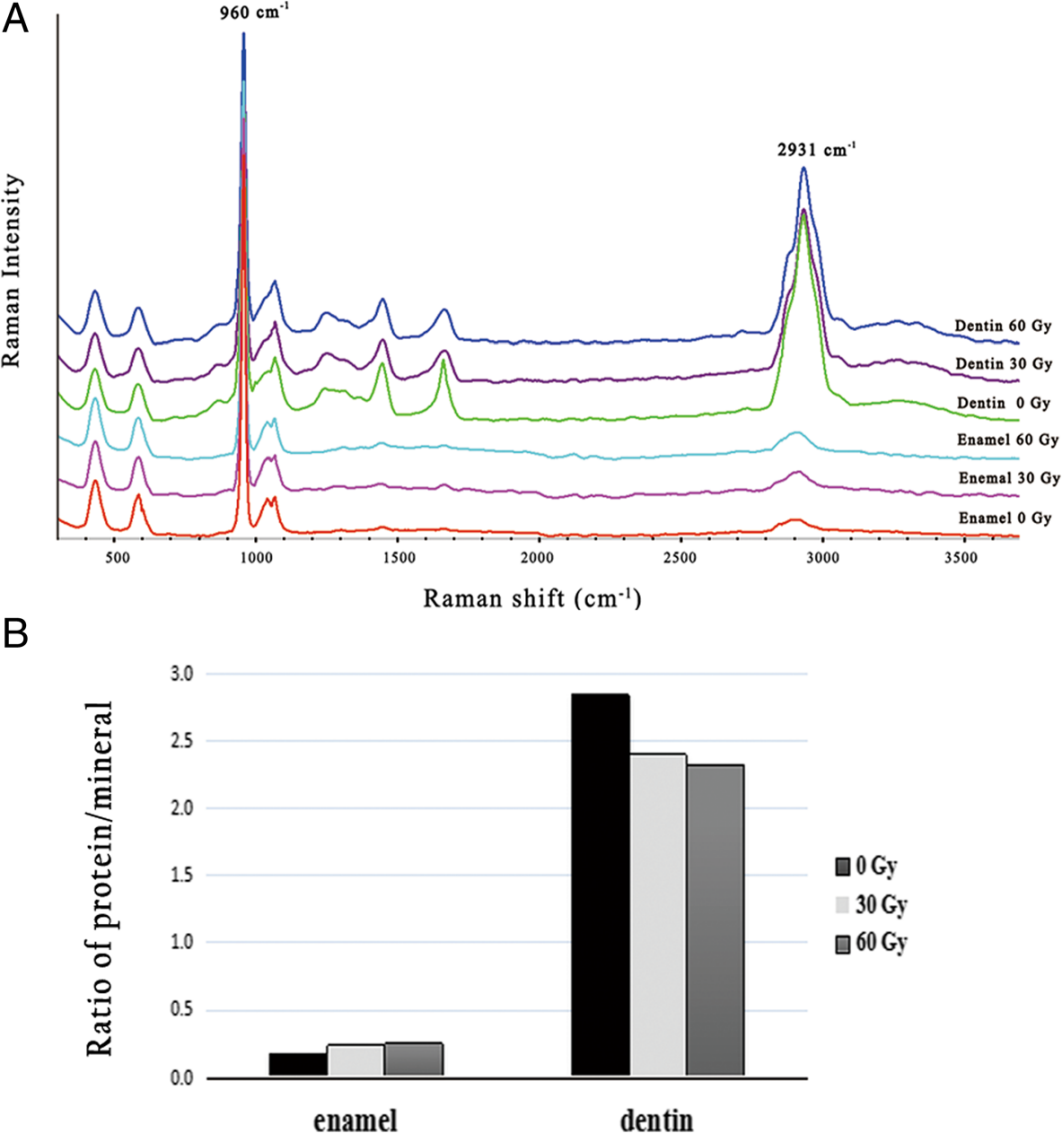
Raman spectral analysis of enamel and dentin in the three groups. **a** Comparison of Raman spectra. **b** Ratios of protein at 2931 cm^− 1^/phosphate at 960 cm^− 1^. With doses increasing, the protein-to-mineral ratio (2931/960 cm^− 1^) in enamel showed a slight increase, while the ratio in dentin decreased

Raman spectra for enamel and dentin after 0-, 30-, and 60-Gy irradiation are presented in Fig. 6, with small differences observed following irradiation. The protein-to-phosphate ratios (2931/960 cm^− 1^) for enamel and dentin in each group are presented. In enamel, the protein-to-mineral ratio (2931/960 cm^− 1^) was gradually raised with increased radiation doses, ranging from 0.174 to 0.256. In contrast, the protein-to-mineral ratio (2931/960 cm^− 1^) in dentin was decreased when doses increased, ranging from 2.843 to 2.324.

## Discussion

Radiotherapy is one of the major methods for the treatment of head and neck cancer. Clinically, a radiation dose of 2 Gy/day was assigned to patients to achieve a cumulative dose of 60 Gy [15]. With the progress in radiation techniques, there are fewer side-effects of radiotherapy [16]. Nevertheless, dentition is still inevitably involved in the targeted areas of irradiation, and radiation-induced damage to dental hard tissue cannot be avoided. Clinical investigations have revealed that the severity of tooth injury is related to the radiation dose to the tooth. Three tiers of dose response were found: From 0 to 30 Gy of radiation resulted in minimal tooth damage. Between 30 and 60 Gy, the tooth dose-damage relationship increased by a factor of 2 or 3. At 60 Gy or more, this relationship increased by a factor of 10 [7]. For that reason, three groups (0 Gy, 30 Gy, and 60 Gy) were studied in the present investigation. Degenerative changes in dental hard tissue were evident after exposure to 30 Gy of radiation. When the dose accumulated to 60 Gy, more destruction of dental hard tissue could be observed, which is in accord with reports from previous studies [10, 12].

For decades, alterations in the mechanical properties of enamel and dentin have been measured by multiple methods. However, there have been few investigations of the DEJ-adjacent region. The DEJ and associated inner enamel play an important role in inhibiting crack propagation and exhibiting higher fracture toughness [17]. Dusevich et al. noticed that an organic enamel layer extending from the DEJ about 50–400 μm into the cuspal enamel, which provided a key biomechanical linkage between the enamel and the DEJ [18]. This was an important consideration when the measurement sites of microhardness and elastic modulus were selected in this study. Apart from two sites at one-half the thickness of buccal enamel and dentin, two sites located 50 μm from the DEJ in enamel and dentin were selected for monitoring of the changes in mechanical properties near the DEJ.

In view of microhardness and elastic modulus values, post-irradiation impairment exists in enamel, dentin, and the DEJ to various degrees. Exposure of 30 Gy could apparently cause reductions in microhardness and elastic modulus of enamel near the DEJ. However, no significant change was found at the sites of middle enamel, middle dentin, and the DEJ-adjacent dentin after an accumulated exposure of 60 Gy. It seems that the DEJ-adjacent region is especially prone to post-irradiation degeneration, which is in accordance with the research conducted by al-Nawas et al. [19]. Reduced microhardness and elastic modulus at the DEJ-adjacent enamel may decrease its ability to resist tooth deformation during mastication [20], resulting in enamel exfoliation several months after radiation. Additionally, we noticed that the values of micro hardness and elastic modulus in the 30 Gy group at some points were slightly lower than that of the 60 Gy group, though the differences were not statistically significant (*p* > 0.05, in Figs. 1b, 2a, Tables 1 and 2). We attribute these diverging results to the individual variation of teeth, and the anisotropy of enamel and dentin [21]. The mechanical properties are influenced not only by the region of tooth but also by the orientation of enamel rod and dentinal tubule. In the present study, although we have taken measures (i.e., inclusion and exclusion criteria, random grouping, section in a bucco-lingual direction, measurement sites selection) to control the differences, the microhardness and elastic modulus values were somehow different from what we supposed. This controversial phenomenon has been observed in several studies [10, 12]. To minimize the random error, larger sample size is advised in the future study.

The results of micro-morphological observation were in accord with those measured by the microhardness tester and AFM. The early destruction of the DEJ-adjacent region after radiation was evident under the SEM and microhardness indentation analyses. The DEJ in the 30-Gy and 60-Gy groups was diffuse, discontinuous, and unstable. As could be seen in the microhardness indentations, fissures were apparent near the DEJ in the 30-Gy and 60-Gy groups, and most of the fissures appeared in enamel, in agreement with the theory that the DEJ-adjacent region is especially prone to post-irradiation degeneration. In addition, AFM revealed that the post-irradiation destruction of the interprismatic enamel substance was more obvious compared with that of the enamel prism. The increased content of organics in the interprismatic region may contribute to the damage [22]. The impairment of the interprismatic region will weaken the connection between apatites, leading to the occurrence of micro-cracks and a rough enamel surface [23].

Differences in the mechanical roles of and morphologic changes in enamel, dentin, and the DEJ may arise from their different internal structures and organic and mineral compositions [24]. In the present study, we characterized the crystal properties of enamel and dentin following radiation exposure through XRD. It is well-known that the major phase of dental hard tissue is hydroxyapatite. In addition to Ca_10_(PO_4_)_6_(OH)_2_, there is a considerable amount of calcium-deficient apatite, which is referred to as Ca_10-x_(HPO_4_)_x_(PO_4_)_6-x_(OH)_2-x_. This kind of calcium-deficient apatite is less stable than Ca_10_(PO_4_)_6_(OH)_2_. When demineralization occurs, calcium-deficient apatite is easily dissolved, leading to reduced crystallinity. Based on XRD analysis, such reduced crystallinity is more distinct than dentin, suggesting more radiation-induced damage to the apatite in enamel. As a result, the enamel would be more vulnerable to acid attacks than would intact enamel, and the biomechanical properties appear to be more affected [19, 25].

The results of Raman spectroscopy and EPMA show the post-irradiation changes in mineral and protein composition. An increase in the phosphate/organics ratio around the DEJ after radiotherapy was found by Read et al., indicating loss of organics at the DEJ-adjacent region [26]. In the present study, it is interesting to note that the changes in the mineral and protein components of enamel and dentin were different. In enamel, the protein-to-mineral ratio (2931/960 cm^− 1^) was slightly increased with increasing doses, suggesting that the loss of mineral was more obvious. On the contrary, the ratio of 2931/960 cm^− 1^ in dentin was decreased, implying more loss of protein. In addition, by area-mapping using EPMA for the components of Ca and P over the fissure near the DEJ, we found the loss of Ca and P to be obvious in the fissure. The Ca/P ratio in the fissure was higher than that of intact enamel, suggesting the loss of calcium-deficient apatite in the fissure. Considering the post-irradiation disintegration of calcium-deficient apatite, the use of fluoride products may be beneficial for the prevention of radiation caries in patients post-irradiation.

This article is based on an ex-vivo study, which attempts to distinguish direct radiation-induced effects on dental hard tissue from tooth damage associated with xerostomia. The results of our study do have confirmed the tooth damage induced by radiation. However, there are some limitations with this study. Firstly, we are unable to simulate the attenuation effect of the jaw bones and oral soft tissues to radiation. The teeth in the oral cavity are inferred to receive a smaller dose than the clinical treatment dose. Thus, when discussing clinical dose-relative effect, the attenuation effect of surrounding tissue should be taken into consideration. What’s more, as a multifactorial disease, radiation caries can be influenced by the oral ecosystem (e.g., differences in salivary flow, microbial composition, dietary changes) in post-irradiated patients. In the present ex-vivo study, we could not evaluate the effects of those factors on the onset and progression of radiation caries. In-vivo studies are needed to better investigate the interaction between the radiation-induced damage on dentition and the radiation-induced changes of oral ecosystem.

In this study, we assessed the direct radiation-induced effects on dental hard tissue through multiple methods. As already indicated, changes in mechanical properties, micro-morphology, crystal properties, and chemical composition were evident, as reflected in the instability of the DEJ, reductions in microhardness and elastic modulus at enamel near the DEJ, and decreased crystallinity, together with losses in mineral and protein in both enamel and dentin. Based on our results and those of previous articles, it is proposed that the direct effect of radiation on dental hard tissue, coupled with post-irradiation xerostomia, may be causal factors for radiation caries. We speculated on the pathogenic mechanisms of post-irradiation dental hard tissue damage:

1. Radiation interacts with organics and water and induces free radicals and hydrogen peroxide in dental hard tissue [27].
2. Higher contents of organics in the DEJ-adjacent area make it susceptible to radiation [22, 28]. Degeneration of organics weakens the interaction between enamel and the DEJ, resulting in destabilization of the DEJ-adjacent region and impaired mechanical properties [29], which may account for the enamel exfoliation and dentin exposure in teeth post-irradiation.
3. Degeneration of organics and minerals also weakens the interactions of hydroxyapatite crystals, leading to decreased crystallinity of apatite and higher solubility in saliva at low pH. Enamel crystals appeared to be more vulnerable to radiation compared with dentin, which may be one of the reasons explaining the formation of craters in superficial enamel.
4. As a result, defects in the microstructure of dental hard tissue, such as fissures at the DEJ and the porosity of enamel, will be beneficial for the attachment and colonization of bacteria, which, combined with indirect effects induced by radiation [10, 30], increases the risk of caries.

According to the observations of our study and potential risk factors of radiation caries, two prophylactic and restorative treatment recommendations are drawn up for radiologists and dentists:

1. Limit the the amount of radiation that dentition and salivary glands are exposed to, without compromising tumor control probability. Beyond 30 Gy of radiation will cause a permanent damage to both teeth and the glands [7]. To minimize the damage of surrounding normal tissues, oral stents has been used during head and neck radiotherapy for decades [31]. Additionally, with the introduction of intensity-modulated radiotherapy (IMRT) which reduces the irradiated volume by shaping the spatial distribution of radiation to target mainly the tumor [32], it is expected that the incidence of radiation caries will be decreased.
2. Education and practices of oral health care are extremely important for patients undergoing radiotherapy. It should be encouraged to use a soft toothbrush and floss or an interproximal brush to effectively remove dental plaque especially plaque attached at cervical areas. Due to the ability of fluoride ion to combine with apatite forming fluorhydroxyapatite [Ca_10_(PO_4_)_6_(OH)_2-2x_F_2x_], which is harder and less susceptible to dissolution, the use of fluoride becomes an ideal preventive strategy to promote remineralization and inhibit demineralization of tooth surfaces subjected to acids [33]. Daily use of high-concentrated fluoride products such as fluoride mouthwash and sodium fluoride gel was recommended during and after radiotherapy [34].

## Conclusions

In conclusion, we have shown the direct radiation-induced effects on the mechanical properties, micro-morphological structures, crystallinity, and chemical composition of dental hard tissue. The early destruction of the DEJ and DEJ-adjacent enamel, combined with the decreased crystallinity of enamel under radiation exposure, may be related to the formation of characteristic radiation caries.

## Abbreviations

AFM: atomic force microscopy
DEJ: dentino-enamel junction
EPMA: electron probe microanalysis
FWHM: full-width half-maximum
JCPDS: Joint Committee on Powder Diffraction Standards
SEM: scanning electron microscopy
XRD: x-ray diffraction
